# Structures and dynamics of Rpd3S complex bound to nucleosome

**DOI:** 10.1126/sciadv.adk7678

**Published:** 2024-04-10

**Authors:** Chengcheng Wang, Chen Chu, Zhouyan Guo, Xiechao Zhan

**Affiliations:** ^1^ Westlake Laboratory of Life Sciences and Biomedicine, 18 Shilongshan Road, Hangzhou 310024, Zhejiang Province, China.; ^2^ Key Laboratory of Structural Biology of Zhejiang Province, School of Life Sciences, Westlake University, 18 Shilongshan Road, Hangzhou 310024, Zhejiang Province, China.; ^3^ Institute of Biology, Westlake Institute for Advanced Study, 18 Shilongshan Road, Hangzhou 310024, Zhejiang Province, China.

## Abstract

The Rpd3S complex plays a pivotal role in facilitating local histone deacetylation in the transcribed regions to suppress intragenic transcription initiation. Here, we present the cryo–electron microscopy structures of the budding yeast Rpd3S complex in both its apo and three nucleosome-bound states at atomic resolutions, revealing the exquisite architecture of Rpd3S to well accommodate a mononucleosome without linker DNA. The Rpd3S core, containing a Sin3 Lobe and two NB modules, is a rigid complex and provides three positive-charged anchors (Sin3_HCR and two Rco1_NIDs) to connect nucleosomal DNA. In three nucleosome-bound states, the Rpd3S core exhibits three distinct orientations relative to the nucleosome, assisting the sector-shaped deacetylase Rpd3 to locate above the SHL5-6, SHL0-1, or SHL2-3, respectively. Our work provides a structural framework that reveals a dynamic working model for the Rpd3S complex to engage diverse deacetylation sites.

## Teaser

Structures of the Rpd3S complex bound to native nucleosome reveal a dynamic working model to engage diverse deacetylation sites.

## INTRODUCTION

In eukaryotic cells, DNA is packaged in the nucleus by histone and nonhistone proteins into a highly condensed structure termed chromatin with the nucleosome as elemental cornerstone. Chromatin structural variation regulates the accessibility of underlying DNA, via a sophisticated set of posttranslational modifications of histones and dynamic remodeling of nucleosomes (*1*–*3*). Histone deacetylases (HDACs) hydrolytically cleave acetyl-Lys in histones, rendering them as epigenetic targets in cancer, neurodegeneration, inflammation, and metabolic disorders (*4*, *5*). The zinc-dependent class I HDACs play a crucial role in transcriptional repression and epigenetic landscaping (*6*). Nonetheless, they appear to be denied direct access to the chromatin. How might they precisely navigate toward chromatin targets? The answer seems to lie in their collaborative partnership with other proteins. Over the past two decades, a multitude of functional complexes, including SIN3L/S (also called Rpd3L/S), NuRD, CoREST, SMRT, MiDAC, MIER, and RERE, have been identified to contain class I HDACs and involved in diverse cellular process (*7*–*10*). Besides, it is generally accepted that HDACs are able to deacetylate numerous histone substrates and appear to have low specificity (*11*). How can they efficiently target a wide array of acetylation sites? The molecular mechanism of the HDACs working on nucleosomes remains misty.

The Rpd3 of *Saccharomyces cerevisiae* is the earliest identified and extensively studied HDAC, existing in two distinct complexes (Rpd3L and Rpd3S), each with its own organizational mode (*12*–*19*). As the catalytic core within the Rpd3L complex, Rpd3 is directed to promoters (such as INO1, IME2, and SPO13) by the DNA-binding transcription regulators to repress transcription initiation (*20*, *21*). Besides, the Rpd3 also functions globally and deacetylates large contiguous regions in euchromatin (*22*). The Rpd3S complex is implicated in the execution of these tasks and recruited through histone H3K36 di/tri-methylation following RNA polymerase II elongation, rather than DNA binding proteins (*23*–*25*). This complex maintains a hypoacetylated state in the transcribed regions, effectively suppressing cryptic transcription. To explore its natural working mechanism, we assembled the Rpd3S complex with endogenous nucleosomes and resolved the cryo–electron microscopy (cryo-EM) structures in three distinct mononucleosome-bound states. The structures uncover the complex and dynamic nature for the Rpd3S binding to nucleosome.

## RESULTS

### Structure determination of the Rpd3S-nucleosome complex

**Fig. 1.**
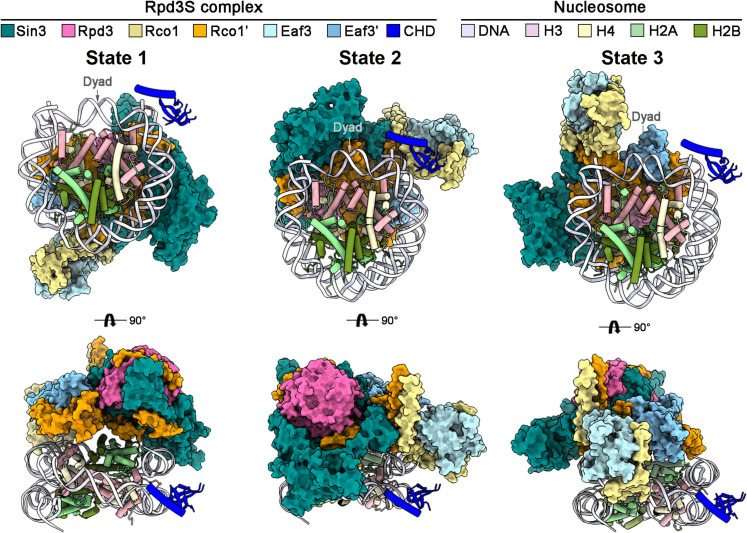
Overall structures of the nucleosome-bound Rpd3S complex in three different states. Cryo-EM structures of the budding yeast Rpd3S complex in three nucleosome-bound states (named state 1, state 2, and state 3, respectively). Two relevant views are shown. The individual protein component of Rpd3S is shown as colored surface except the chromodomain (CHD) of Eaf3 in cartoon. The histones and DNA of nucleosome are shown as colored cartoon. The average resolutions of these EM maps lastly resolved at 2.9 to 3.9 Å. The Eaf3_CHD is at a moderate local resolution in all nucleosome-bound states. All structural images here were generated in ChimeraX (*55*) and PyMOL (*56*).

We purified the endogenous Rpd3S complex from *S. cerevisiae* with all five known subunits by employing a C-terminal Flag-tag on Rco1. To mimic more physiological substrates, we extracted and purified nucleosomes from human embryonic kidney (HEK) 293 T cells, which contained DNA with a persistence length of ~150 base pairs and histones with natural acetylation (fig. S1, A to C). The glycerol gradient centrifugation results show a clear shift of the nucleosome peak, suggesting that the Rpd3S complex binds to the nucleosome (fig. S1, D to F). Cryo-EM analysis yielded a series of reconstructions for the 1:1 Rpd3S-nucleosome complex in different states, and three among them could be lastly resolved at atomic resolutions (named state 1, state 2, and state 3) (Fig. 1, figs. S2 to S5, and table S1). The EM maps allowed protein assignment and de novo atomic modeling covering six molecules for the Rpd3S complex, including Sin3, Rpd3, and two copies each of Rco1 (named as Rco1 and Rco1′) and Eaf3 (named as Eaf3 and Eaf3′). In addition, the structure of the nucleosome core particle (NCP) for each state was generated by fitting the model of nucleosome [Protein Data Bank (PDB) code: 2CV5] (*26*) into the high quality EM maps and further refined.

Structural analysis of the three nucleosome-bound states reveals that the chromodomain (CHD) of Eaf3 binds at the super helix loop 7 (SHL7) in each state (Fig. 1 and fig. S5); beyond that, the core region of Rpd3S, in different states, assumes distinct spatial orientations relative to the nucleosome (Fig. 1), which will be further discussed in the following sections.

### Rigid core of the Rpd3S complex

**Fig. 2.**
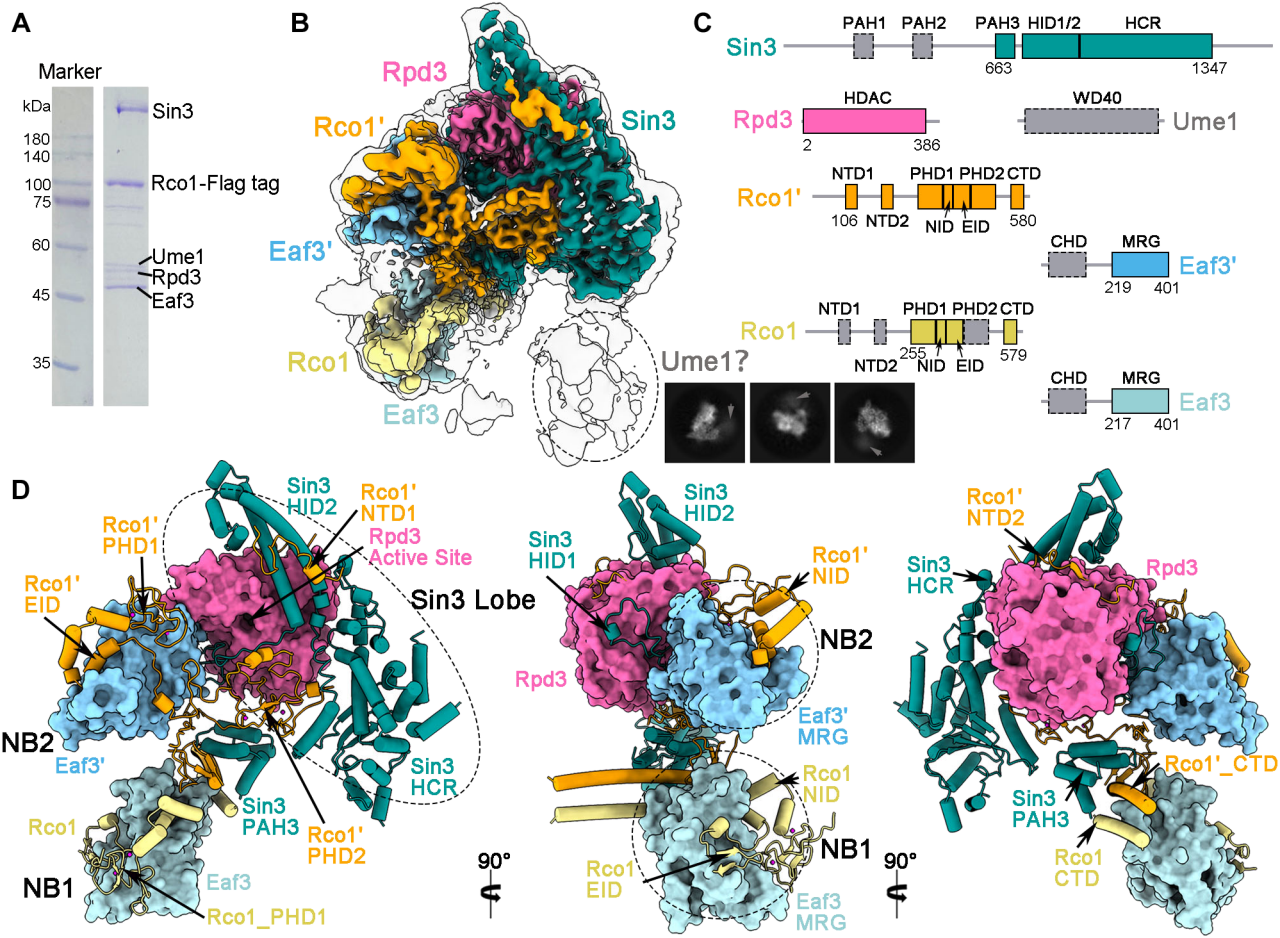
Rigid core of the Rpd3S complex. (**A**) Purification of the *S. cerevisiae* Rpd3S complex. The components of Rpd3S were separated on 12% SDS–polyacrylamide gel electrophoresis and further confirmed by mass spectrometry analysis. (**B**) Overall EM density map of the apo Rpd3S complex with individual component color-coded. Six components are able to be de novo atomic modeled, including Sin3, Rpd3, and two copies each of Rco1 (named as Rco1 and Rco1′) and Eaf3 (named as Eaf3 and Eaf3′). The extra EM map near the Sin3_HCR at a moderate local resolution may belong to the Ume1. The 2D averages of the apo Rpd3S are shown with the potential position of the Ume1 labeled by gray arrow. (**C**) A schematic diagram of the domain organization of modeled subunits in the Rpd3S complex. Unassigned domains are colored gray. (**D**) The structure of the core region of the Rpd3S complex in three different views. The Rpd3S core is almost identical in the apo- and three nucleosome-bound states, indicating that it is a rigid complex. The Rpd3S core reveals a compact architecture composed of three modules: a Sin3 Lobe (Sin3 and Rpd3) and two modules involved in the nucleosome binding (named “NB1,” consisting of Rco1_PHD1/NID/EID and Eaf3_MRG, and “NB2,” consisting of Rco1′_ PHD1/NID/EID and Eaf3′_MRG, respectively).

We also performed cryo-EM analysis for the apo Rpd3S complex and yielded a reconstruction covering six molecules (including two copies each of Rco1 and Eaf3) at an average resolution of 3.3 Å (Fig. 2, A to C, and figs. S6 to S8). The Rpd3S core reveals a compact architecture composed of three modules: a Sin3 Lobe (Sin3 and Rpd3) and two modules involved in the nucleosome binding (named “NB1” and “NB2,” respectively) (Fig. 2, C and D). The multi-domain subunits Sin3 and Rco1 play important roles in the organization and structural integrity of the rigid core. The Sin3, highly conserved in eukaryotes, consists of three paired amphipathic α helix domains (PAH1/2/3), a HDAC interaction domain (HID), and a C-terminal highly conserved region (HCR; residues 926 to 1347). The Rco1′, assigned in our structures, contains two N-terminal domains (NTD1/2; touching the Sin3_HID2 or Rpd3, respectively), two plant homeodomains (PHD1/2), a nucleosome interacting domain (NID; residues 311 to 332), a Eaf3 interacting domain (EID; residues 333 to 376), and C-terminal domain (CTD; connecting the Sin3_PAH3 and NB1 module). The Sin3_HID/HCR and Rco1′_NTD wrap around the HDAC Rpd3 (Fig. 2D). The Sin3_PAH3 catches an α helix of Rco1′_CTD. Two copies each of the Rco1_EID interacts with the Eaf3_MRG, forming the NB1 and NB2, respectively. Of note, the NB1 and NB2 are asymmetrical. The Rco1′_PHD1 in the NB2 is close to the active site of Rpd3, while the Rco1_PHD1 in the NB1 is far away. The central positioning of Rco1′_PHD2 within the complex serves to bridge the Sin3 Lobe and NB1/2, making a contribution to the stabilization of the Rpd3S core (Fig. 2D).

In both the nucleosome-free/bound states, the conformation of the Rpd3S core composed by the Sin3 Lobe and NB1/2 remains almost identical, suggesting its structural rigidity. Besides, extra EM map near the Sin3_HCR at a moderate local resolution may belong to the Ume1 (Fig. 2B). The PAH1/2 of Sin3 and the NTD/PHD2 of Rco1 are unable to be modeled (Fig. 2, B and C), likely due to their inherent flexibility. In addition, two copies of Eaf3_CHD are not assigned in the apo state; nevertheless, in all nucleosome-bound states, either of the two is stabilized by the nucleosome at SHL7 (fig. S5).

Rpd3S complex is widely existed in eukaryotes (fig. S9A). The advent of the structure of Rpd3S complex allows its comparison with that of the SIN3S complex from *Schizosaccharomyces pombe* (*sp*SIN3S) (*18*) and the human SIN3B complex (*17*). The structural features of *sc*Rpd3S and *sp*SIN3S exhibit similarity in the view that oriented toward the nucleosome, comprising the deacetylases, Sin3 homologs, and NB1/2 [also referred to as HB1/2 in prior studies (*18*)]. Conversely, the structures show divergence in the rear sides (fig. S9B), likely attributed to the limited sequence similarity between Rco1 and Cph1/2. Furthermore, the structure of the human SIN3B complex resembles that of the Rpd3S core, with the exception that the NB1 is absent (fig. S9C) (*17*). This observation implies the conservation of working mechanisms within this family of complexes across eukaryotes.

### Interaction between the Rpd3S core and nucleosomal DNA

**Fig. 3.**
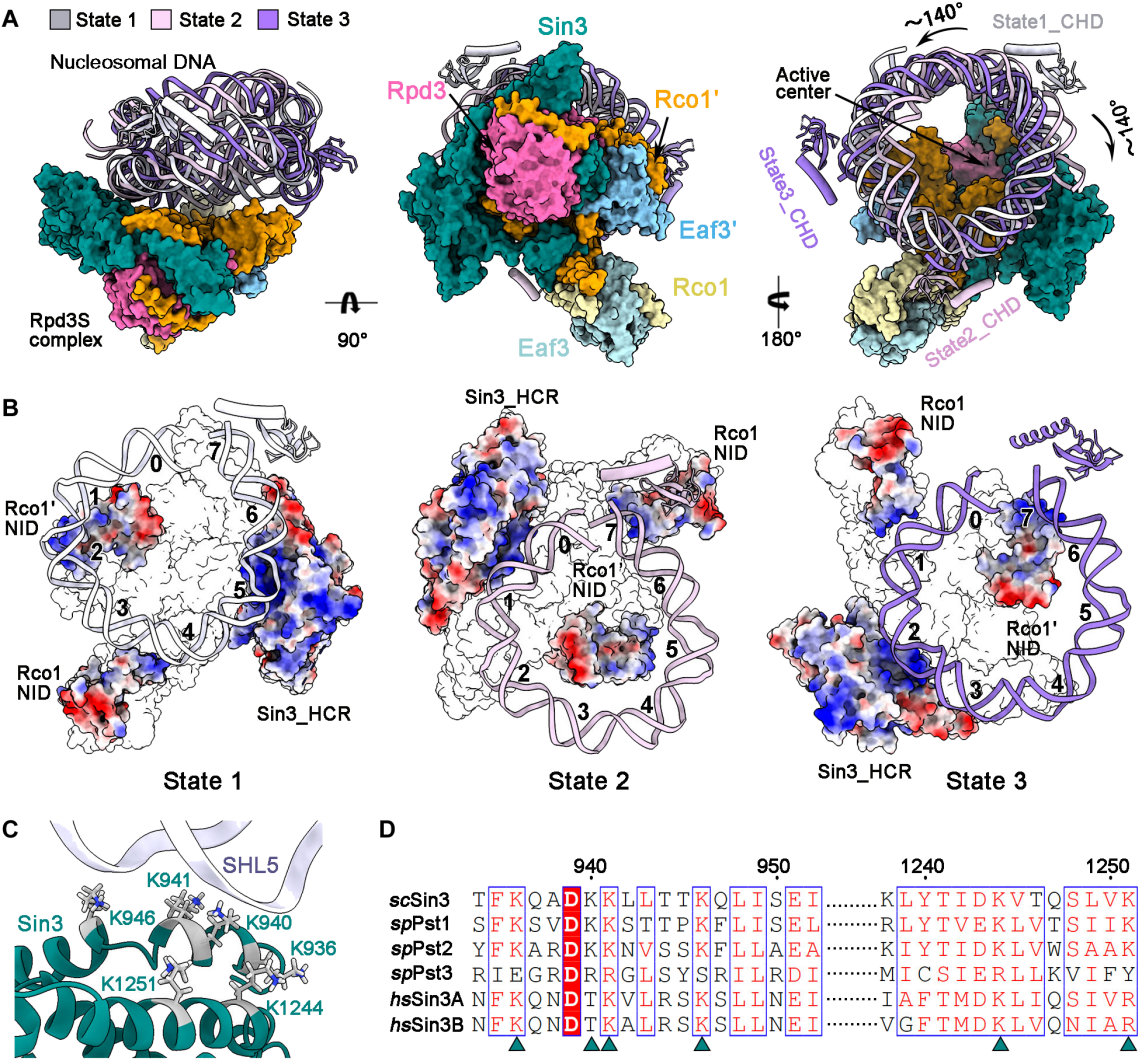
Three anchors of the Rpd3S core well accommodate the nucleosomal DNA. (**A**) Structure comparison among the three nucleosome-bound Rpd3S complex. The structures are superposed by the Rpd3S core, which is shown as color-coded surface. The Eaf3_CHDs coupled with nucleosomal DNAs in state 1, 2, or 3 are shown as cartoon colored gray, pink, and purple, respectively. (**B**) The interaction between three positive-charged anchors of Rpd3S and nucleosomal DNA. The Sin3_HCR, Rco1′_NID, and Rco1_NID, shown electrostatic potential map, bind at SHL5/4/2 in state 1, respectively (left); at SHL1/5/7 in state 2, respectively (middle); and at SHL2/7/1 in state 3, respectively (right). (**C**) The interface between Sin3_HCR and nucleosomal DNA at SHL5 in state 1. (**D**) The sequence alignment of the Sin3 homologs from different species. *sp*, *Schizosaccharomyces pombe*; *sc*, *Saccharomyces cerevisiae*; *hs*, *Homo sapiens*. The residues Lys^936^, Lys^940^, Lys^941^, Lys^946^, Lys^1244^, and Lys^1251^ of Sin3, highly conserved in eukaryotes, provide a positive-charged surface for interaction with DNA.

A comprehensive structural analysis has been conducted to elucidate the general characteristics for the Rpd3S core binding to nucleosome. When superposed by the Rpd3S core, the nucleosome in state 2 or state 3 undergoes a rotation of ~140° clockwise or counterclockwise from state 1, respectively (Fig. 3A). The Sin3_HCR and two Rco1_NID within the Rpd3S core offer three positive-charged anchors that involve the interaction with nucleosomal DNA. In detail, the Sin3_HCR, Rco1′_NID and Rco1_NID bind at SHL5/4/2 in state 1, respectively (Fig. 3B, left); at SHL1/5/7 in state 2, respectively (Fig. 3B, middle); and at SHL2/7/1 in state 3, respectively (Fig. 3B, right). Notably, the residues Lys^936^, Lys^940^, Lys^941^, Lys^946^, Lys^1244^, and Lys^1251^ of Sin3_HCR, highly conserved in eukaryotes, provide a positive-charged surface for the interaction with DNA (Fig. 3C).

In summary, the Rpd3S core presents three anchoring points to define a circle, precisely accommodating the disc-shaped mononucleosome. Nevertheless, the interaction between these anchors and DNA appears inadequate to achieve stabilization in a specific state, necessitating the involvement of additional factors.

### Structure and location of the deacetylase Rpd3 relative to nucleosome

**Fig. 4.**
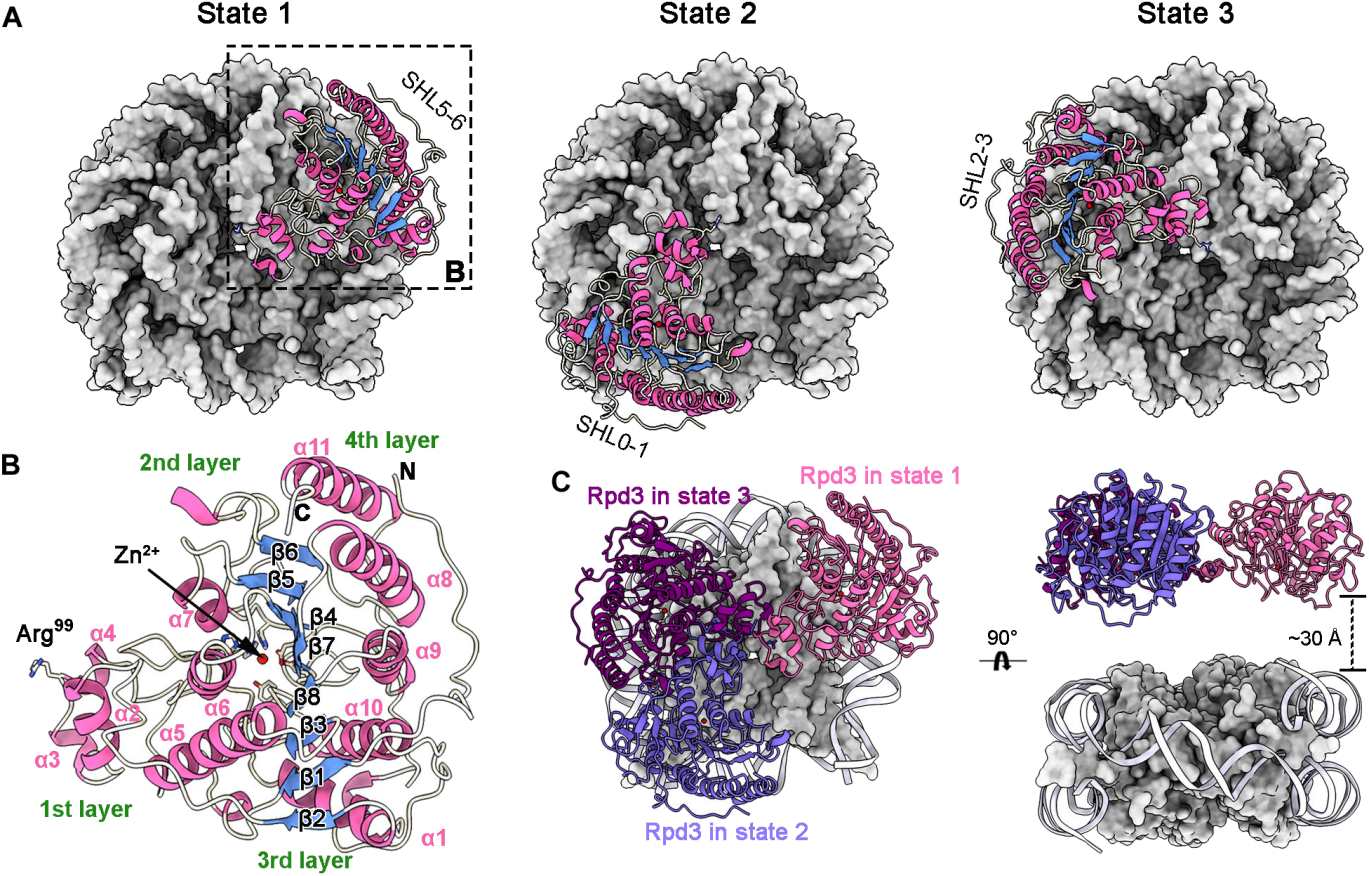
Structure of the deacetylase Rpd3 relative to nucleosome. (**A**) Structure of the deacetylase Rpd3 relative to nucleosome in state 1, 2, or 3 in the top view. The Rpd3 takes different orientation in three states and stays overhead the SHL5-6, SHL0-1, or SHL2-3, respectively. (**B**) The sector-shaped structure of the *S. cerevisiae* Rpd3, with the residue Arg^99^ as the vertex, about 48 Å in radius, and about 60° in arc radian, has four layers. The fourth layer comprises five helices (α1, 8-11), which locates at the periphery of nucleosome. The active site is located between the third layer (β1-8) and the second layer (α5-7). The first layer, consisting three helices (α2-4), is overhead the center of the nucleosome. (**C**) The orientation of the Rpd3 in state 1, 2, or 3 superposed by the nucleosome. The Rpd3 in state 1, 2, or 3 is colored pink, slate blue, and dark orchid, respectively. Two different views are shown. The Rpd3 has no direct interaction with nucleosome and has been placed in the same plane ~30 Å away from the nucleosome with the assistance of the auxiliary subunits.

The *S. cerevisiae* Rpd3 belongs to the Zn^2+^-dependent class I HDAC family, showing classical overall fold composed of an eight-stranded parallel β sheet flanked by several α helices on both sides (*27*). The purified recombinant class I HDACs lack individual access to chromatin, resulting in their enzymatic inactivity (*11*). In this work, structural analysis reveals that the Rpd3 has no direct interaction with nucleosome. Instead, aided by the auxiliary subunits, the Rpd3 is located above SHL5-6, SHL0-1, or SHL2-3 in state 1, 2, or 3, respectively (Fig. 4A). Notably, the structural depiction of Rpd3 in relation to the nucleosome resembles a sector, with the pivotal residue Arg^99^ serving as the vertex (Fig. 4B). This sector spans ~48 Å in radius, covering an arc of ~60°. Moreover, the sector-shaped structure can be segmented into four distinct layers. The fourth layer encompasses five helices (α1, 8-11), positioned above the outer edge of the nucleosome. Worth highlighting is that both the N terminus and C terminus, which encompass phosphorylation sites, reside within this fourth layer and remain exposed to the surrounding space. The active site is situated between the third layer (β1-8) and the second layer (α5-7). The first layer, consisting three helices (α2-4), is positioned directly above the nucleosome’s central region. Upon nucleosome superposition, the deacetylases in all states are located at the same plane 30 Å away from the nucleosome, potentially engaging in a revolving motion around the vertex of the sector (Fig. 4C).

### Potential deacetylation site for each state

**Fig. 5.**
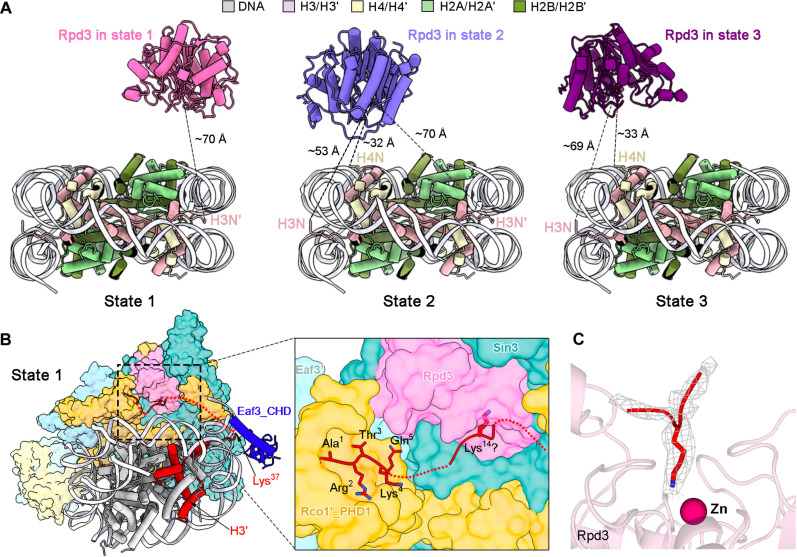
Structure analysis of the potential deacetylation site in state 1, 2, or 3. (**A**) Structures of the Rpd3-nucleosome in state 1, 2, or 3. The nucleosome is displayed in the same view, with the distance from the active site of Rpd3 measured in each state. In state 1, only the H3′ tail extended from SHL7/-1 in its fully extended conformation can satisfy the distance to the active site of the Rpd3. However, in states 2 and 3, the corresponding distance allows to bind with several lysine residues on H3 or H4 N terminus. (**B**) Structure analysis reveals the candidate substrate of Rpd3 in state 1. Around the active site of Rpd3, a fragment from the residues 1 to 5 of H3 binds the Rco1′_PHD1, which is consistent with the previous reports. This structure feature together with the distance between the N-terminal tailed-up residue and the active site enables us to assign the target as the residue Lys^14^ of H3′ in state 1. (**C**) EM density of the peptide in the pocket of the active site in state 1.

The enzymatic activity of the *S. cerevisiae* Rpd3S complex on histone H3 Lys^14/18/23/27^ and H4 Lys^5/8/12^ residues has recently been confirmed (*19*, *28*). Fortunately, in this study, three distinct assemblies of Rpd3S-nucleosome complexes have been successfully captured. Subsequent structural analysis has been conducted to predict the potential deacetylation sites for each state. We systematically quantified the distance from the active site to the N-terminal tailed-up residue of each histone in state 1, 2, or 3, respectively (Fig. 5 and table S2). In state 1, only the H3′ tail extended from SHL7/-1 in its fully extended conformation can satisfy the distance to the active site of the Rpd3 (Fig. 5A, left). Notably, in state 1, the Rco1′_PHD1 adjacent to the active center captures a peptide, likely corresponding to residues 1 to 5 of histone H3 (Fig. 5B and fig. S10). It is consistent with the previous reports that the PHD1 of Rco1 recognizes the unmodified N terminus of H3 (*29*–*31*). Furthermore, our cryo-EM analysis reveals an extra density for a peptide with a long sidechain extended into the pocket of the active site in state 1 (Fig. 5C). Together, it is most likely that the residue Lys^14^ of H3′ has been griped in the active center of state 1. However, in states 2 and 3, the corresponding distance allows to bind with several lysine residues on H3 or H4 N-terminal tails (Fig. 5A, middle and right, and table S2).

## DISCUSSION

In contrast to the majority of class I HDAC complexes (such as Rpd3L, NuRD, MiDAC, and MIER1), which comprise two copies of deacetylase (*15*, *19*, *32*–*34*), the Rpd3S complex consists of a solitary deacetylase, presenting a more compact subunit arrangement. Here, we unveil the cryo-EM structures of the Rpd3S complex in both nucleosome-free and three nucleosome-bound states, revealing that its exquisite architecture properly accommodates a mononucleosome. The Rpd3S core provides three positive-charged anchors to interact with nucleosomal core DNA. In addition, one copy of the Eaf3_CHD, which suggested to recognize the H3K36me (*24*, *35*, *36*), binds at the SHL7 at a moderate local resolution (fig. S5). However, there is no obvious EM density beside the other side, which might be caused by the methylation level of natural nucleosomes. It indicates that at least one copy of H3K36me2/3 is necessary for the Rpd3S complex to target nucleosome. Furthermore, our structural analysis reveals the EM maps corresponding to a peptide within the active site pocket in each state. We suppose that the tension generated by the deacetylase tugging the N terminus of histones contributes to reinforcing the interaction between the Rpd3S and the nucleosome. Collectively, the recognition and binding of the Rpd3S complex to the nucleosome necessitate a convergence of multiple factors.

Upon comparing the structures in three nucleosome-bound states, the rigid core of Rpd3S takes different orientations relative to nucleosome. Besides, except these three models reconstructed at atomic resolutions, our sample seems to contain other binding forms for Rpd3S-nucleosome but at low resolutions based on cryo-EM analysis (fig. S3). Furthermore, the elaborate structure analysis reveals that the Rpd3S core likely rotates around an axle relative to the center of nucleosome (movie S1). It suggests that the Rpd3S complex facilitates the deacetylase in achieving the precise position relative to the nucleosome, thereby enabling it function at distinct deacetylation sites. The variable interaction modes for Rpd3S and nucleosome augment the flexibility and applicability of the deacetylation system. Of note, the nucleosomal DNA exhibits deviations from a straightforward circular structure due to its left-handed helix configuration. Consequently, the actual motion trajectory of the Rpd3S complex is expected to be more intricate than our simulation.

Previous reports indicated that the presence and length of the linker DNA could affect Rpd3S function (*25*). This work illuminates that the linker DNA was not a necessary condition for nucleosome recruiting Rpd3S. During the preparation and peer review of this manuscript, several groups also solved the cryo-EM structures of Rpd3S-nucleosome complex using recombinant nucleosomes (*28*, *37*, *38*). Most of them have used a similar recombinant nucleosome with H3K36MLA (mimicking the H3K36me3) and extra linker DNA to trap in one similar conformational state, exhibiting similarities to our state 3 but slight alterations in orientation (fig. S11A). Comparison between the structures of state 3 in this work and the Close/Loose_State (PDB code: 7YI4/7YI5) from Guan *et al*. (*28*), the Rpd3S core exhibits a discernible motion of gradually sliding away from the nucleosome. The Sin3_HCRs anchor on the SHL2s. Nevertheless, the NB2 is sliding from SHL7 to the linker DNA; and the NB1 is moving away the nucleosome, likely leading to its flexibility and low resolution in Close/Loose_State. Previous reports revealed that the Rpd3S complex could also bind di-nucleosome (*39*). It indicates that the linker DNA may mediate the Rpd3S to work on neighbor nucleosomes (fig. S11B). Besides, Dong *et al*. (*40*) reported a conformation state (Rpd3S_NCP 167 bp/MLA) similar with our state 1, consistent with our opinion that H3K36me triggers recruiting of Rpd3S to nucleosome but may not infect the conformational change during deacetylation.

In summary, the structural analysis in this work points to a potential working model for the Rpd3S complex using “three anchors” to slide along the DNA track (including nucleosome core DNA and linker DNA) to engage different deacetylation sites (fig. S12). The Rpd3S/SIN3S complex is prevalent among eukaryotes. Although the structures of the *S. cerevisiae* Rpd3S complex and the *S. pombe* SIN3S complex are not entirely identical, the three-anchor architecture exhibits remarkable similarity (fig. S9), implying that the sliding working model could be applicable to other species as well.

Our previous work presented the structures of the SIN3S and SIN3L complexes in *S. pombe* and revealed their similarities and differences in assembly mechanism (*18*). Comparing the structures of these complexes in *S. cerevisiae*, we also find that the Sin3 Lobe of the Rpd3S is much similar with the ARM2 of the Rpd3L (fig. S13A) (*15*, *16*). Furthermore, we try to see whether the nucleosome binding mechanism of Rpd3S could partially extend to the Rpd3L complex. We superposed the Rpd3L to the Rpd3S in three nucleosome-binding states by Sin3, revealing the potential working model for its ARM2; while the ARM1 is away from current nucleosome, suggesting its function on the linker DNA or −1/+1 nucleosome (fig. S13B). Apart from the interaction between the Sin3 and nucleosome, many other factors (such as the H3K4me3 signal and specific DNA sequence) may also associate in the genomic recruiting the Rpd3L complex.

## MATERIALS AND METHODS

### The *S. cerevisiae* strain

The *S. cerevisiae* strain used to purify the Rpd3S complex carries a 3x-Flag tag (DYKDHDGDYKDHDIDYKDDDDK) at the C terminus of Rco1 protein. The DNA sequences for 3x-Flag tag and a HphMX6 marker were amplified by polymerase chain reaction (PCR) from the plasmid pF6Aa-C3FLAG-HphMX6. The PCR product was transformed into a wild-type (*trp*-) *S. cerevisiae* strain by the lithium acetate method (*41*). Transformants were selected on hygromycin-YPD (yeast extract, peptone, and dextrose) solid medium. Correct integration of the 3x-Flag tag was further confirmed by PCR and Western blot.

### Purification of the apo Rpd3S complex

Following procedures previously described in (*18*), the Rco1-tagged culture was grown on 1× YPD medium for 8 to 10 hours at 30°C to an optical density at 600 nm of ~6. The collected cell pellets were washed with buffer containing 1 mM phenylmethylsulfonyl fluoride (PMSF) and resuspended in lysis buffer containing 50 mM Hepes-NaOH (pH 7.4), 350 mM NaCl, 15% glycerol (v/v), and protease inhibitors [1 mM PMSF, aprotinin (2.6 μg/ml), pepstatin (1.4 μg/ml), and leupeptin (5 μg/ml)]. The cell suspension was dropped into liquid nitrogen to form yeast beads and pulverized to powder in a Retsch ZM200 nitrogen mill. The frozen yeast powder was thawed at room temperature and resuspended in the same lysis buffer. The cell lysate was first centrifuged at 12,000 rpm for 30 min, and the supernatant was centrifuged again at 12,000 rpm for another 1 hour. The resulting supernatant was loaded into the ANTI-FLAG M2 resin (Sigma-Aldrich) and eluted using elute buffer containing FLAG peptide (DYKDDDDK;0.5 mg/ml), 20 mM Hepes-NaOH (pH 7.4), 150 mM NaCl, and 5% glycerol (v/v). The eluate was concentrated and then applied for glycerol density gradient centrifugation. The glycerol gradient was prepared with light buffer containing 10% glycerol (v/v), 20 mM Hepes-NaOH (pH 7.4), 50 mM NaCl, and 2 mM dithiothreitol (DTT) and with heavy buffer containing 30% glycerol (v/v), 20 mM Hepes-NaOH (pH 7.4), 50 mM NaCl, and 2 mM DTT. After centrifugation at 30,000 rpm for 20 hours at 4°C using a Beckman SW32Ti rotor, the peak fractions were verified further cross-linked by 1 mM bis (sulfosuccinimidyl) suberate (BS3) on ice for 2 hours and quenched by 50 mM tris-HCl (pH 7.4), followed by dialysis for 12 hours against the buffer containing 20 mM Hepes-NaOH (pH 7.4), 50 mM NaCl, and 2 mM DTT. The protein concentration of the apo Rpd3S complex applied to cryo-grid preparation was about 0.1 mg/ml.

### Nucleosome preparation from HEK 293T cells

We prepared the endogenous nucleosome form HEK 293T cells using the nucleosome preparation kit (Active Motif) according to the previous report (*42*). In short, HEK 293T cells were harvested from the 10-cm plate with 70 to 80%. Then, cells were washed with 1× phosphate-buffered saline buffer twice and resuspended in the ice-cold lysis buffer supplemented with protease inhibitor cocktail and 100 mM PMSF. After incubation on ice for 30 min, the lysed cells were centrifuged at 2400*g* for 10 min at 4°C to isolate the nuclei. The nuclei pellet was resuspended in the digestion buffer supplemented with protease inhibitor cocktail and PMSF. After incubation at 37°C for 5 min, the resuspended nuclei were mixed with diluted enzymatic shearing cocktail and incubated at 37°C for 1 hour. During the incubation, the mixture was vortexed approximately every 2 min. Last, ice-cold 0.5 M EDTA was added into the mixture for 10 min on ice. Then, the mixture was centrifuged at 21,000*g* for 10 min. Nucleosomes in the supernatant were purified using a Superose 6 increase 10/300 GL column (Cytiva) with the running buffer containing 20 mM Hepes-NaOH (pH 7.4) and 50 mM NaCl (fig. S1).

### Pan-deacetylation assay

The deacetylation reactions were performed in a reaction buffer containing 20 mM Hepes-NaOH (pH 7.4), 50 mM NaCl, and 5 mM MgCl_2_. Rpd3S complex (0.1 μM) and endogenous nucleosomes (1 μM) were added and incubated at 30°C for 10, 30, 60, 120, or 180 min, respectively. The reactions were quenched by adding SDS loading buffer and boiled for 10 min. The reaction mixtures were loaded to an 18% SDS–polyacrylamide gel electrophoresis gel and subsequently analyzed by Western blot using the acetylated-lysine antibody (Cell Signaling Technology, no. 9441S).

### Sample preparation for the Rpd3S-nucleosome complex

To assemble the Rpd3S-nucleosome complex for cryo-EM grid preparation, the purified Rpd3S complex and nucleosome was preincubated at a ratio of 1:1 at 4°C for 30 min. The complex was applied to glycerol gradient centrifugation with chemical crosslinking. The glycerol gradient was prepared with light buffer containing 10% glycerol (v/v), 20 mM Hepes-NaOH (pH 7.4), 50 mM NaCl, and 2 mM DTT and with heavy buffer containing 30% glycerol (v/v), 20 mM Hepes-NaOH (pH 7.4), 50 mM NaCl, 2 mM DTT, and 0.1% glutaraldehyde. The samples were centrifuged at 38,000 rpm for 16 hours at 4°C using a Beckman SW41 Ti rotor (fig. S1, E and F). Subsequently, fractions containing cross-linked complexes were quenched by adding 100 mM Tris-HCl (pH 7.6). Peak fractions were pooled and concentrated to about 1 ml, followed by dialysis for 8 hours against a buffer containing 20 mM Hepes-NaOH (pH 7.4), 50 mM NaCl, and 2 mM DTT. The protein concentration of the complexes applied to cryo-grid preparation was about 0.4 mg/ml.

### EM data collection

Uranyl acetate (2% w/v) was used for negative staining. Briefly, 4-μl aliquots of the cross-linked sample at the concentration of 0.05 mg/ml were applied to the glow-discharged copper grid supported by a thin layer of carbon film (Zhongjingkeyi Technology Co. Ltd) for 1 min. The negative stained samples were imaged on a Thermo Fisher Talos L120C TEM microscope operating at 120 kV. The glow-discharged copper Lacey carbon grids (TED PELLA) and Quantifoil gold R1.2/1.3 grids were used for cryo-EM specimen preparation of apo Rpd3S and Rpd3S-nucleosome complex, respectively. Cryo-grids were prepared using Vitrobot Mark IV (FEI Company) operating at 8°C and 100% humidity. After waiting for 1 min, a 4-μl sample was blotted and rapidly plunged into liquid ethane cooled by liquid nitrogen.

Following procedures previously described in (*18*), cryo-EM specimens were imaged on a 300-kV Titan Krios electron microscope (FEI Company) with a normal magnification of ×81,000. Movies were collected by a Gatan K3 direct electron detector equipped with a GIF Quantum energy filter (slit width 20 eV) at the super-resolution mode. A total of 13,348 and 10,633 micrographs were automatically recorded using AutoEMation II (*43*) (developed by J. Lei) and EPU (Thermo) with a defocus range from −1.8 to −2.3 μm for the apo and nucleosome-bound Rpd3S complex, respectively. Each stack of 32 frames was aligned and summed using MotionCor2 with a binning factor of 2, resulting in a pixel size of 1.077 and 1.087 Å for the apo and nucleosome-bound Rpd3S complex, respectively (*44*). Dose weighting was performed concurrently. The defocus value for each image was determined by Gctf (*45*).

### Cryo-EM data processing for the Rpd3S-nucleosome complex

The cryo-image data processing steps for the Rpd3S-nucleosome complex are briefly described here (fig. S2). In total, about 5.3 million particles were generated from 10,625 good micrographs using Gautomatch (developed by K. Zhang; www2.mrc-lmb.cam.ac.uk/download/gautomatch-053/). Multiple rounds of 2D classifications were applied for all the 4× binned particles (pixel size, 4.348 Å), and the good particles were selected for the initial three-dimensional (3D) model generation and further processing. After 2D selection, about 2.7 million 2× binned particles (pixel size, 2.174 Å) were applied for 3D classifications and resulted in about 2.0 thousand good particles. These particles were further applied to local 3D classifications, and three different states of the Rpd3S-nucleosome complex were found. After auto-refinement in Relion (*46*), the three states (state 1, state 2, and state 3) were reconstructed at average resolutions of 3.9, 3.6, and 4.4 Å using 422,198, 327,408, and 213,127 unbinned particles (pixel size, 1.087 Å), respectively. To further improve the local map quality, we applied the soft masks for the Rpd3S and nucleosome parts and performed local classifications. Last, for the state 1, the Rpd3S and nucleosome parts were both refined at average resolutions of 2.9 Å; for the state 2, the Rpd3S and nucleosome parts were reconstructed at average resolutions of 3.6 and 2.9 Å, respectively; and for the state 3, the Rpd3S and nucleosome parts were reconstructed at average resolutions of 3.9 and 3.2 Å, respectively (fig. S3 and table S1).

### Cryo-EM data processing for the apo Rpd3S complex

The cryo-image data processing procedures for the apo Rpd3S complex is briefly presented (fig. S4). For the apo Rpd3S complex, all steps were mainly carried out using RELION 3.0 (*46*) except that is specially mentioned. In total, 12,986 good micrographs were manually selected from 13,348 collected micrographs, and about 2.7 million particles were auto-picked using Gautomatch (developed by K. Zhang; www2.mrc-lmb.cam.ac.uk/download/gautomatch-053/). Multiple rounds of 2D classifications were applied for all the 2× binned particles (pixel size, 2.154 Å), and the initial 3D model of the Rpd3S complex was generated using the select good particles. After 2D selection, about 1.6 million particles were applied for 3D classifications and resulted in about 684 thousand unbinned good particles (pixel size, 1.077 Å). These particles were further applied to multi-reference guided 3D classifications, and, last, about 378 thousand good particles yielded a reconstructions at average resolution of 3.3 Å after nonuniform refinement in cryoSPARC v4 (fig. S5 and table S1) (*47*).

Reported resolutions mentioned above were calculated on the basis of the Fourier shell correlation (FSC) at 0.143 criterion (figs. S3, A to C, and S5A) (*48*). Before visualization, all EM maps were postprocessed and sharpened by applying a negative B-factor in cryoSPARC (*47*). Local resolution variations were estimated for all the EM maps using cryoSPARC (figs. S3, D to F, and S5B) (*47*). The angle distributions of the particles used in the final reconstructions are reasonable (figs. S5C and S6).

### Model building and refinement

The atomic model of the *S. cerevisiae* Rpd3S complex was built de novo on the basis our high-resolution EM maps using Coot (*49*). We first placed the poly-Ala sequences into the EM maps and successfully assigned the different components under the guidance of the individual predicted structure from the AlphaFold database (*50*). Then, the individual reliable domains of the predicted structures of each component were fitted into the EM map using Chimera (*51*) and manually adjusted in Coot. The linker sequences were built de novo on the basis of the clear features of bulk residues.

The atomic models for the Rpd3S-nucleosome complex were generated on the basis of the coordinates of the apo Rpd3S complex and the human nucleosome (PDB code: 2CV5) (*26*). For each state, the structures of the Rpd3S complex and human nucleosome were fitted into the local EM map, which were aligned on the basis of the overall reconstruction, using Chimera (*51*). For the Rpd3S part, the local structure variations were carefully checked and adjusted. For the nucleosome part, the extended flexible region without available EM density map was deleted.

The final atomic coordinates of the *S. cerevisiae* apo Rpd3S and Rpd3S-nucleosome complexes were refined according to the respective EM maps using PHENIX in real space, and secondary structure restraints were generated meanwhile (*52*). Overfitting of the model was monitored by refining the model in one of the two independent maps from the gold-standard refinement approach and testing the refined model against the other (figs. S3, G to I, and S5) (*53*). The structures of the apo Rpd3S and Rpd3S-nucleosome complexes were validated through examination of the Molprobity scores and statistics of the Ramachandran plots (table S1). Molprobity scores were calculated as described (*54*).
