# Supplementary material for: Structures and dynamics of Rpd3S complex bound to nucleosome
Source: Sci Adv. 2024 Apr 10;10(15):eadk7678. doi: 10.1126/sciadv.adk7678 (PMC11006229; doi:10.1126/sciadv.adk7678)
Supplement: Supplementary file 1 — Figs. S1 to S13 Tables S1 and S2 Legend for movie S1 [file sciadv.adk7678_sm.pdf]

Science Advances
AAAS

Supplementary Materials for

Structures and dynamics of Rpd3S complex bound to nucleosome

Chengcheng Wang *et al.*

Corresponding author: Chengcheng Wang, wangchengcheng@westlake.edu.cn;
Xiechao Zhan, zhanxiechao@westlake.edu.cn

Sci. Adv. **10**, eadk7678 (2024)
DOI: 10.1126/sciadv.adk7678

The PDF file includes:

Figs. S1 to S13
Tables S1 and S2
Legend for movie S1

Other Supplementary Material for this manuscript includes the following:

Movie S1

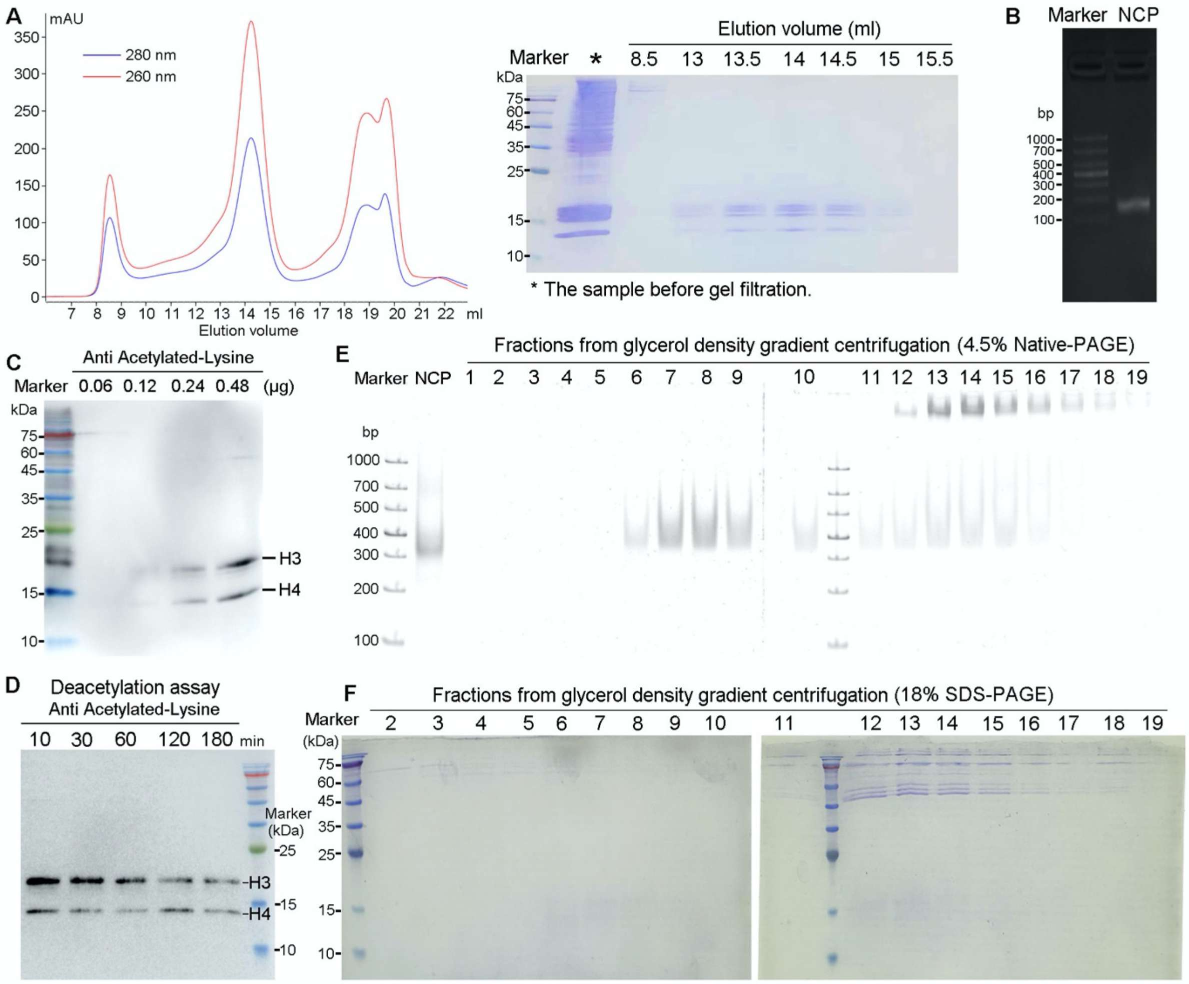

fig. S1 Sample preparation of the Rpd3S-nucleosome complex.

(**A**) Gel filtration analysis of the nucleosome extracted from HEK293T cells. The peak fractions from gel filtration were visualized on SDS-PAGE by Coomassie blue staining. (**B**) SYBR™ Gold-stained agarose gel (1.5%), showing the DNA (about 150 bp) form the purified nucleosomes. (**C**) Anti acetylated-lysine western blot, showing the total acetylation of the purified nucleosomes. The antibody (Cell Signaling Technology, #9441 S) is used in the western blot. (**D**) Pan-deacetylation activity for the Rpd3S using the native nucleosomes as substrates. (**E** and **F**) The peak fractions from glycerol density gradient centrifugation were visualized on native-PAGE by SYBR™ Gold (**E**) and SDS-PAGE by Coomassie blue staining (**F**). Fractions 12-16 were collected for cryo-EM sample preparation.

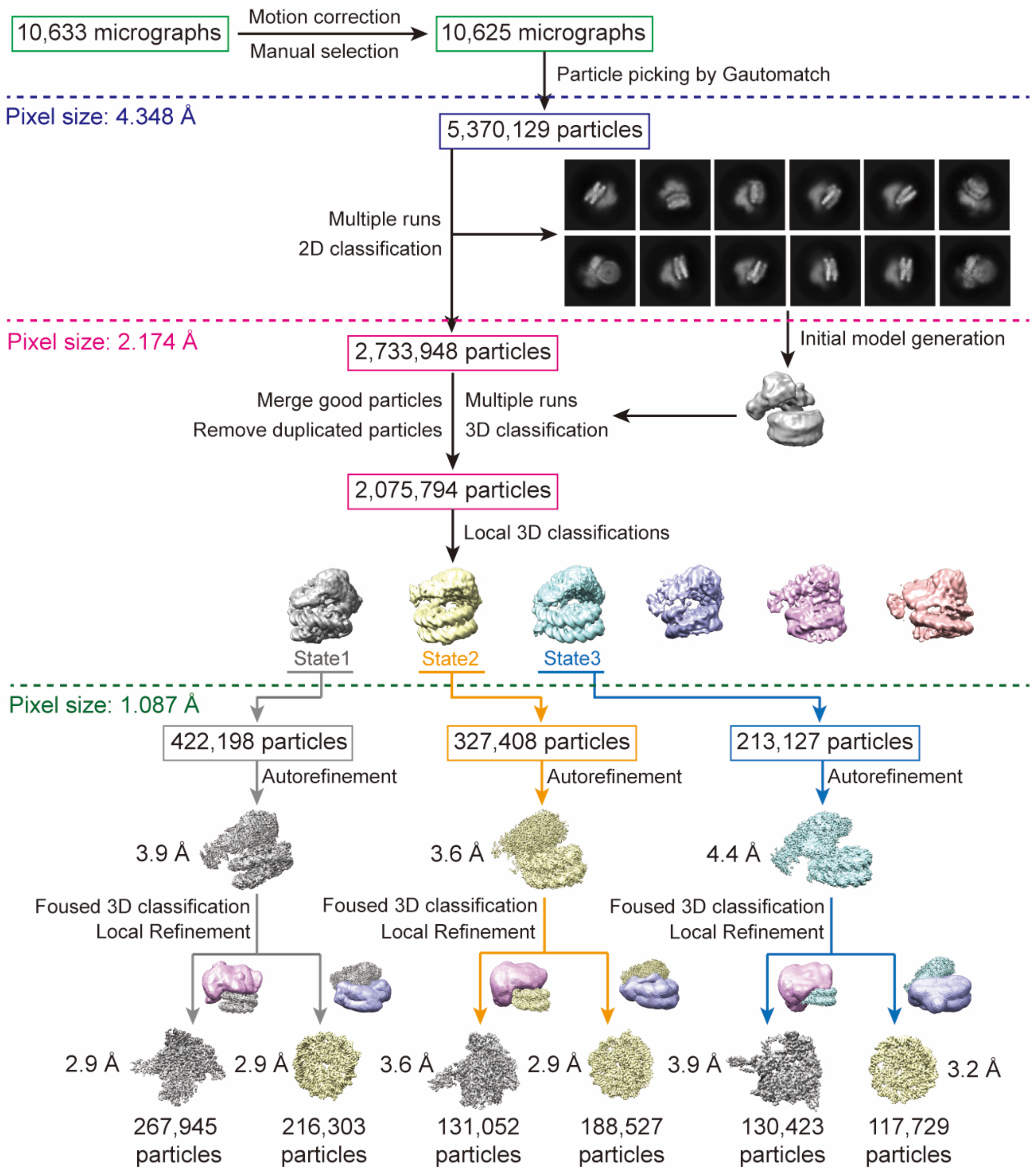

fig. S2 A flow chart of cryo-EM data processing for the Rpd3S-nucleoosme complexes. All processing steps were carried out in RELION 3.0 and cryoSPARC. Please refer to Methods for details.

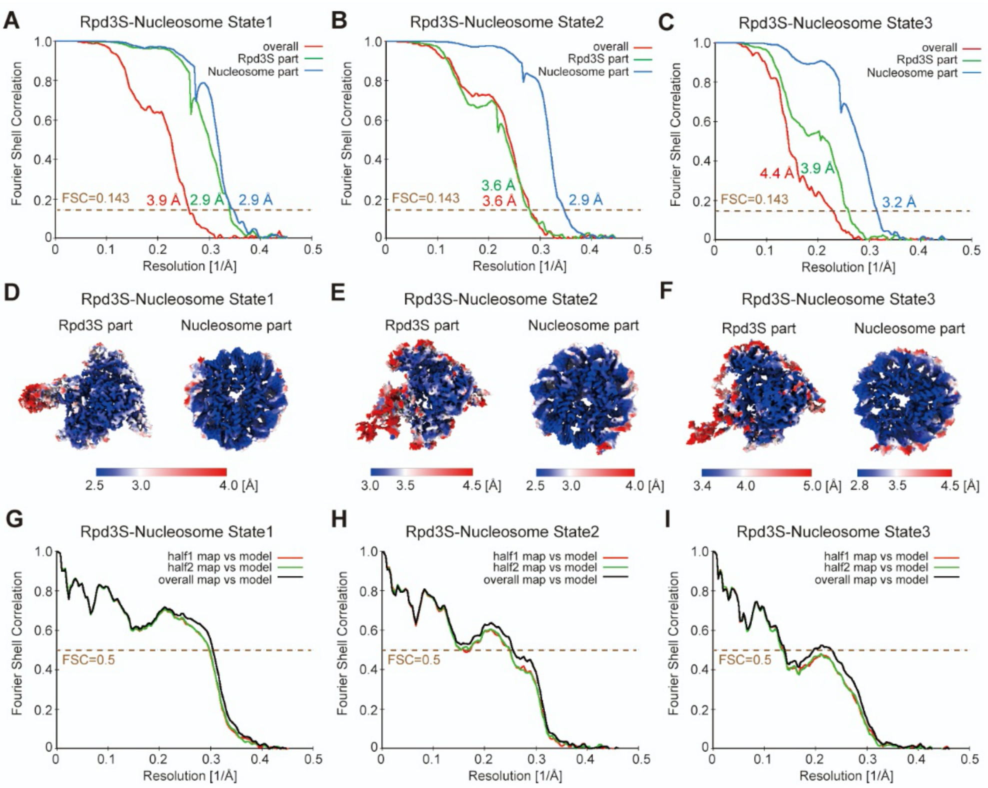

fig. S3 Cryo-EM analysis of the Rpd3S-nucleosome complexes.

(**A**) The final reconstruction for the State 1 of Rpd3S-nucleosome complex displays average resolutions of 3.9 Å, 2.9 Å and 2.9 Å for overall, Rpd3S and nucleosome parts on the basis of the FSC value of 0.143, respectively. (**B**) The final reconstruction for the State 2 of Rpd3S-nucleosome complex displays average resolutions of 3.6 Å, 3.6 Å and 2.9 Å for overall, Rpd3S and nucleosome parts on the basis of the FSC value of 0.143, respectively. (**C**) The final reconstruction for the State 3 of Rpd3S-nucleosome complex displays average resolutions of 4.4 Å, 3.9 Å and 3.2 Å for overall, Rpd3S and nucleosome parts on the basis of the FSC value of 0.143, respectively. (**D-F**) The local resolutions are color-coded for Rpd3S and nucleosome parts of Rpd3S-nucleosome complex in State 1 (**D**), State 2 (**E**) and State 3 (**F**). (**G-I**) The FSC curves for cross-validation between the model and the cryo-EM maps of the Rpd3S-nucleosome complex in State 1 (**G**), State 2 (**H**) and State 3 (**I**). Shown here are the FSC curves between the final refined atomic model and the map from all particles (black), between the model refined in the map from only half of the particles and the map from that same half (red), and between that same model and the map from the other half of the particles (green).

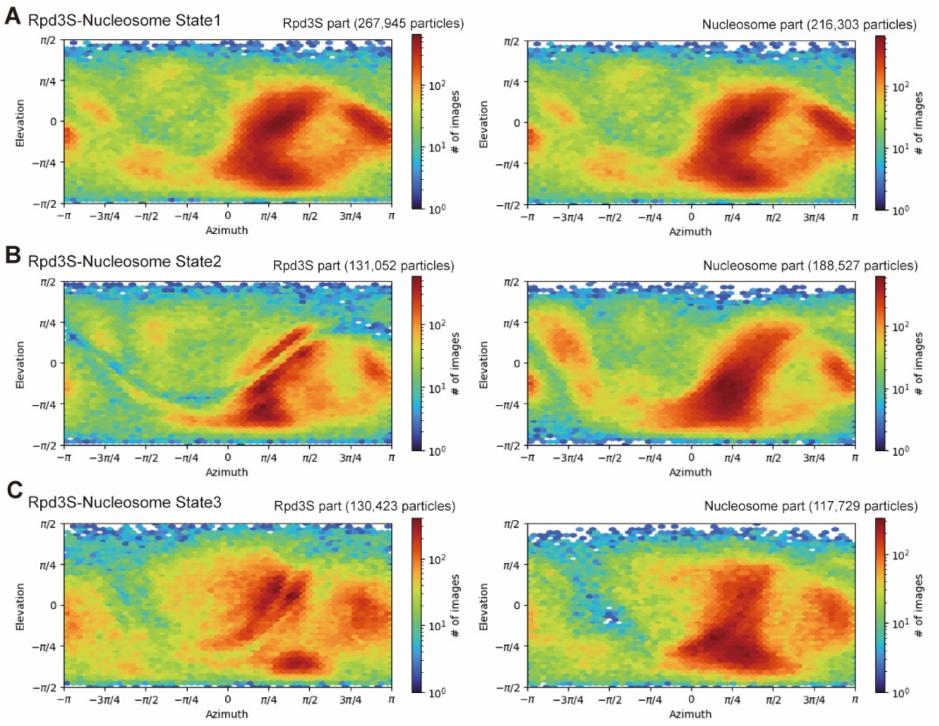

fig. S4 Angular distribution of the particles used for the reconstruction of the Rpd3S-nucleosome complexes.

The angular distribution of the particles used for the reconstruction of the Rpd3S-nucleosome complex in State 1 (**A**), State 2 (**B**) and State 3 (**C**).

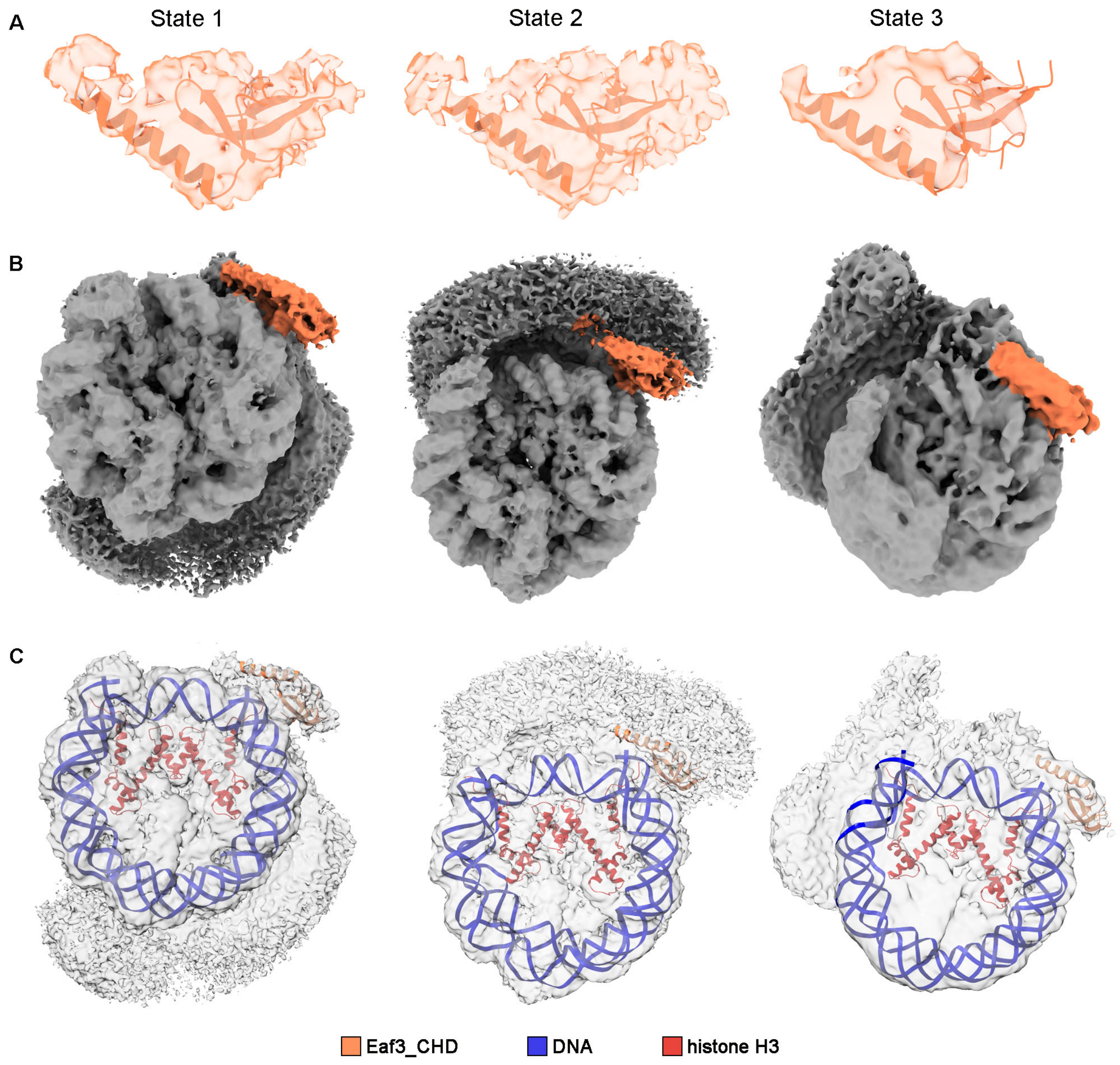

fig. S5 Cryo-EM maps of the Eaf3_CHD in different nucleosome-bound states.
(**A**) Split EM maps of the Eaf3_CHD from three different nucleosome-bound states. (**B**) Either of the two copies of Eaf3_CHD is at a moderate local resolution in nucleosome-bound states. The map of CHD is highlighted in orange, and the rest part is colored in grey. (**C**) Either of the two copies of Eaf3_CHD interacts with the nucleosome DNA.

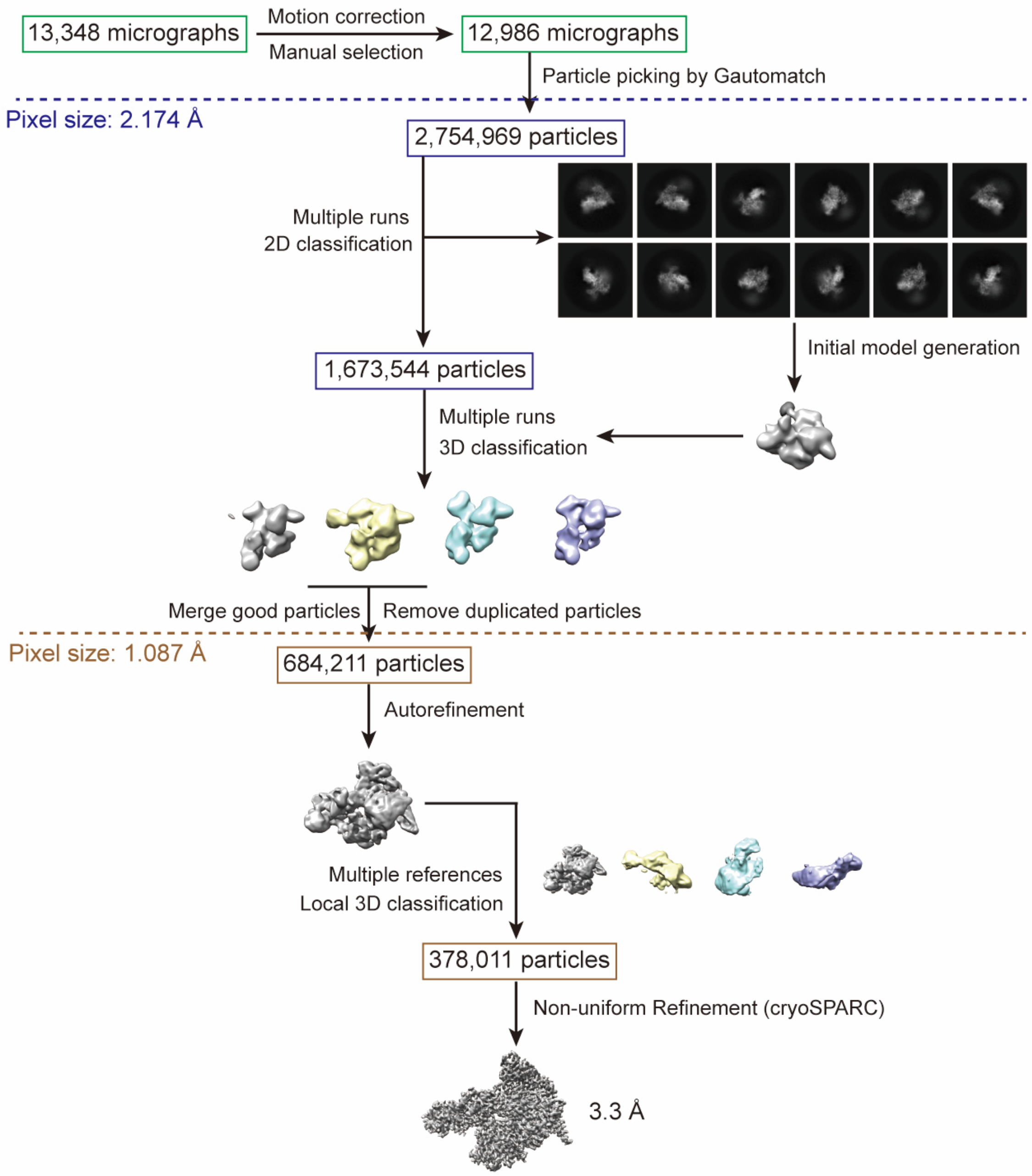

fig. S6 A flow chart of cryo-EM data processing for the apo Rpd3S complex.
All processing steps were carried out in RELION 3.0 and cryoSPARC. Please refer to Methods for details.

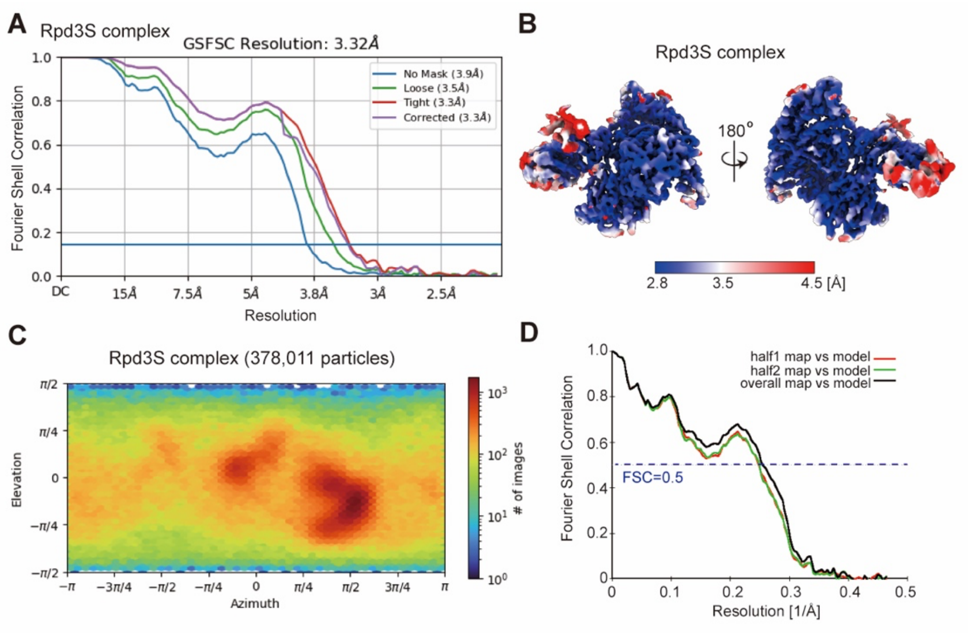

fig. S7 Cryo-EM analysis of the apo-Rpd3S complex.

(**A**) The final reconstruction for the Rpd3S complex displays an average resolution of 3.3 Å on the basis of the FSC value of 0.143. (**B**) Two overall views of the EM density map. The local resolutions are color-coded for different regions of the Rpd3S complex. (**C**) Angular distribution of the particles used for the reconstruction of the Rpd3S complex. (**D**) The FSC curves for cross-validation between the model and the cryo-EM maps of the Rpd3S complex.

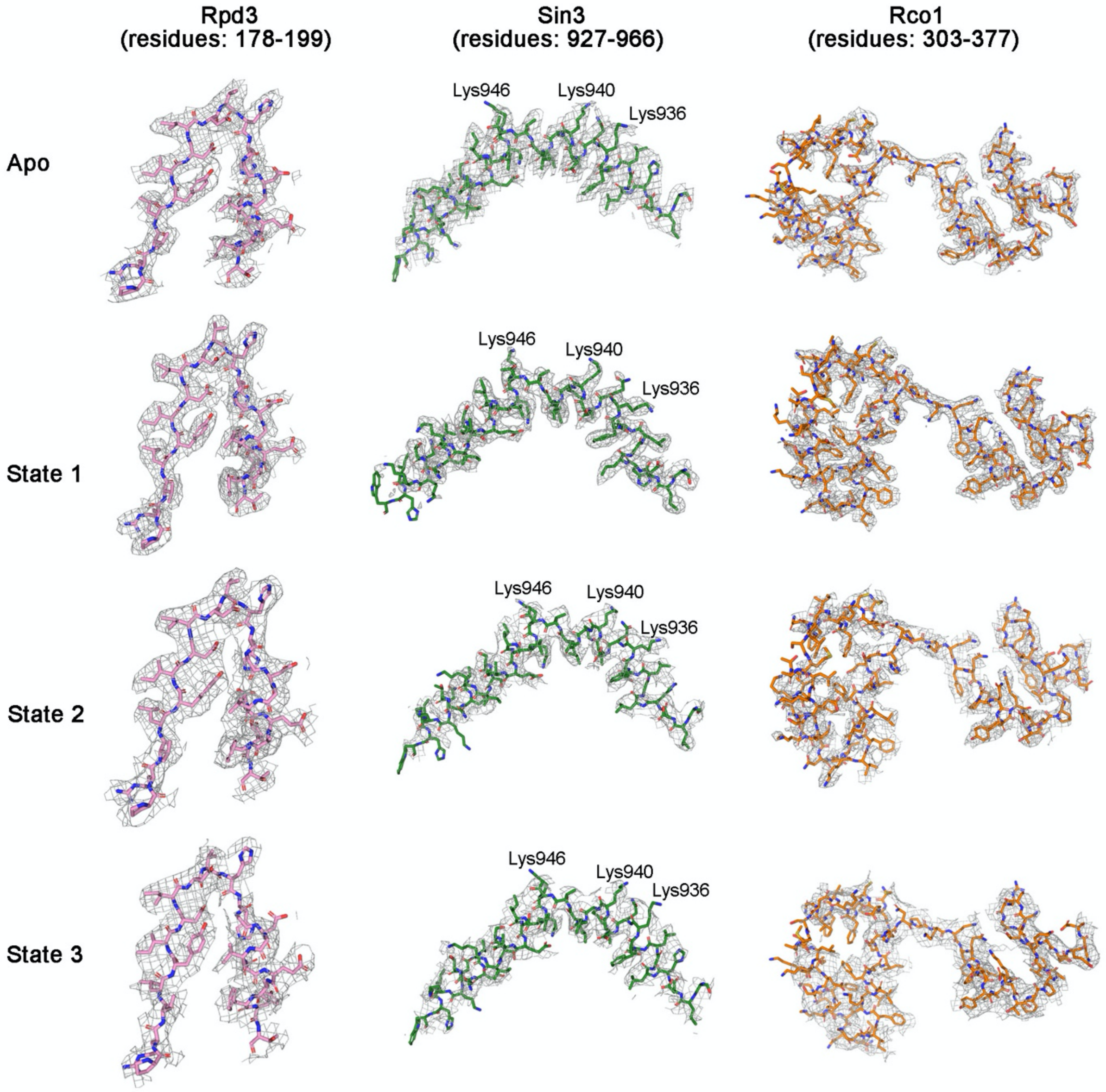

fig. S8 Representative EM maps of the Rpd3S complex in the apo and three nucleosome-bound states.

Close-up views for representative fragments of the colored Rpd3S subunits with EM maps shown as gray meshes. The side chains of representative bulk residues shown as sticks that are used to validate the sequence assignment are labeled. The EM maps were shown in PyMOL and contoured at 5 σ.

A

S. cerevisiae	*S.pombe*	*Homo sapiens*	Characteristic
Sin3	Pst2	SIN3B	Sin3 homolog
Rpd3	Clr6	HDAC1, HDAC2	Class I histone deacetylase
Ume1	prw1	RBBP4, RBBP7	Contain WD40 domian
Rco1	Cph1, Cph2	PHF12	Contain PHD
Eaf3	Eaf3	MORF4L1	Contain CHD and MRG

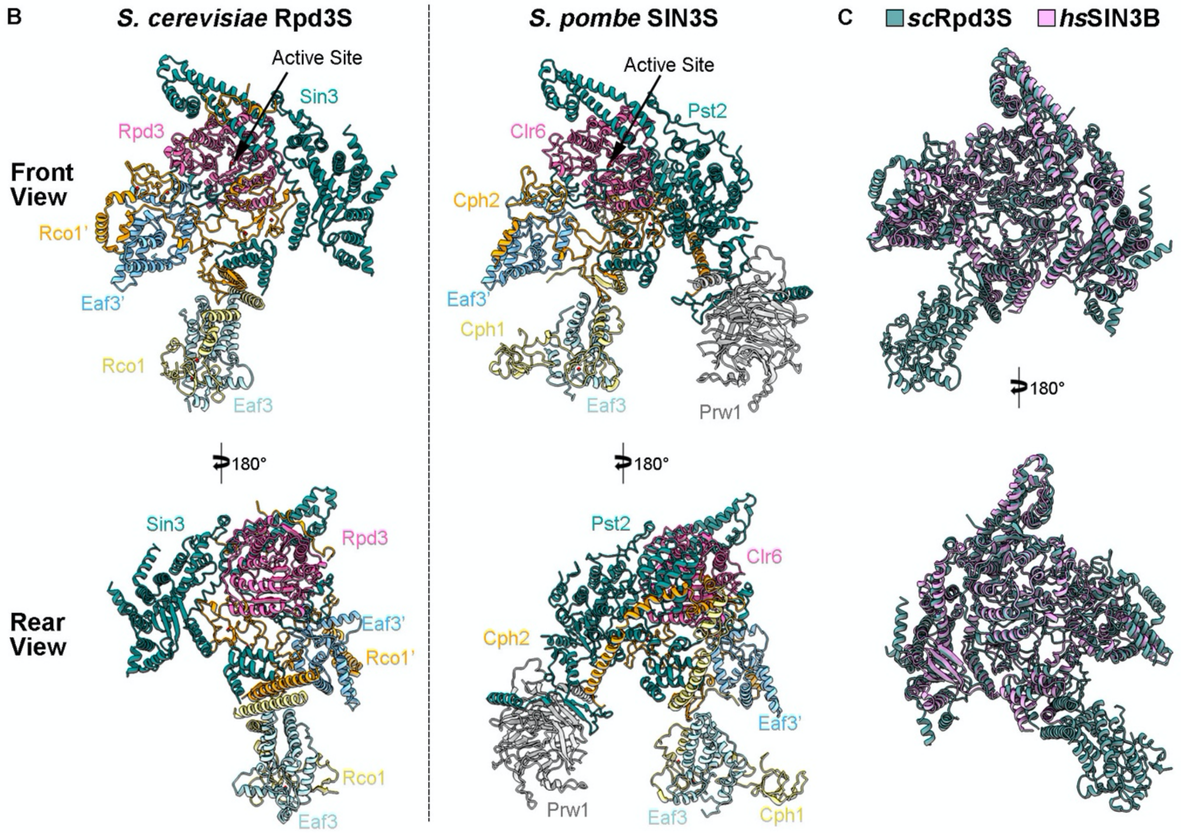

fig. S9 Structural comparison of Rpd3S core in different species.
(**A**) Subunit organization of the Rpd3S complex in different species. (**B**) Structural comparison between *sc*Rpd3S and *sp*SIN3S (PDB code: 8I02). The structures of *sc*Rpd3S and *sp*SIN3S facing to nucleosome (except the WD40-containing protein) are identical , while their rear sides are apparently different. (**C**) Structural comparison between *sc*Rpd3S and *hs*SIN3B. The structure of human SIN3B complex (PDB code: 8BPA) is also similar to that of the Rpd3S except that its NB1 is missing.

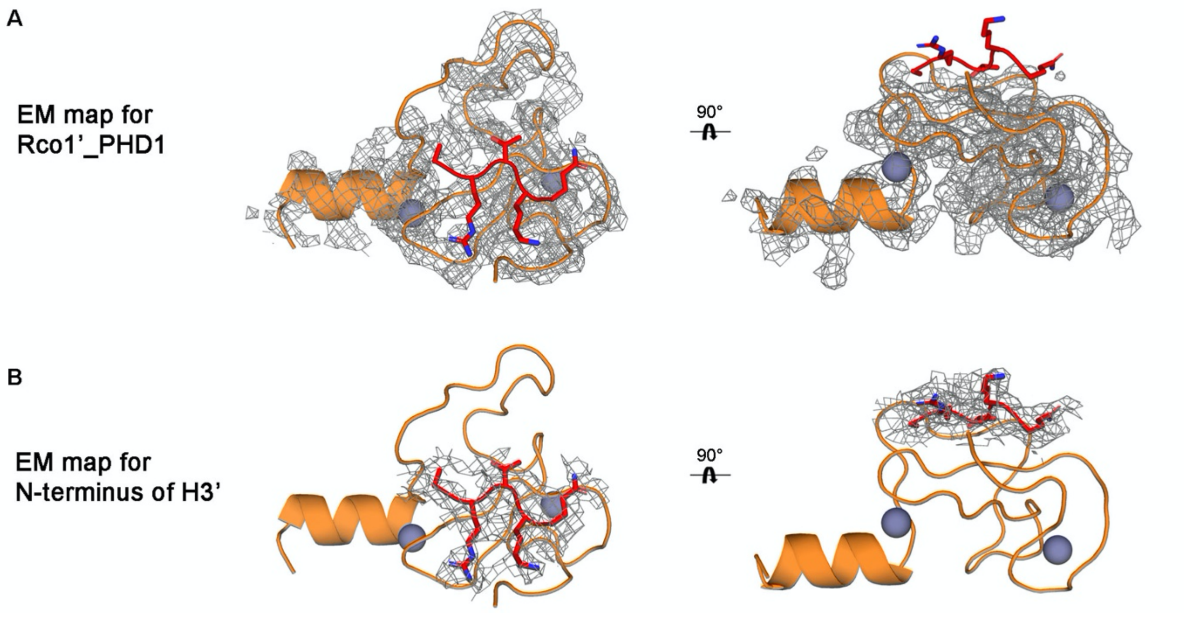

fig. S10 EM maps of the Rco1'_PHD and N-terminal residues of histone H3 in State 1. Close-up views for the Rco1'_PHD (**A**) and the N-terminal residues (from Ala1 to Gln5) of histone H3' (**B**) in State 1 with EM maps shown as gray meshes.

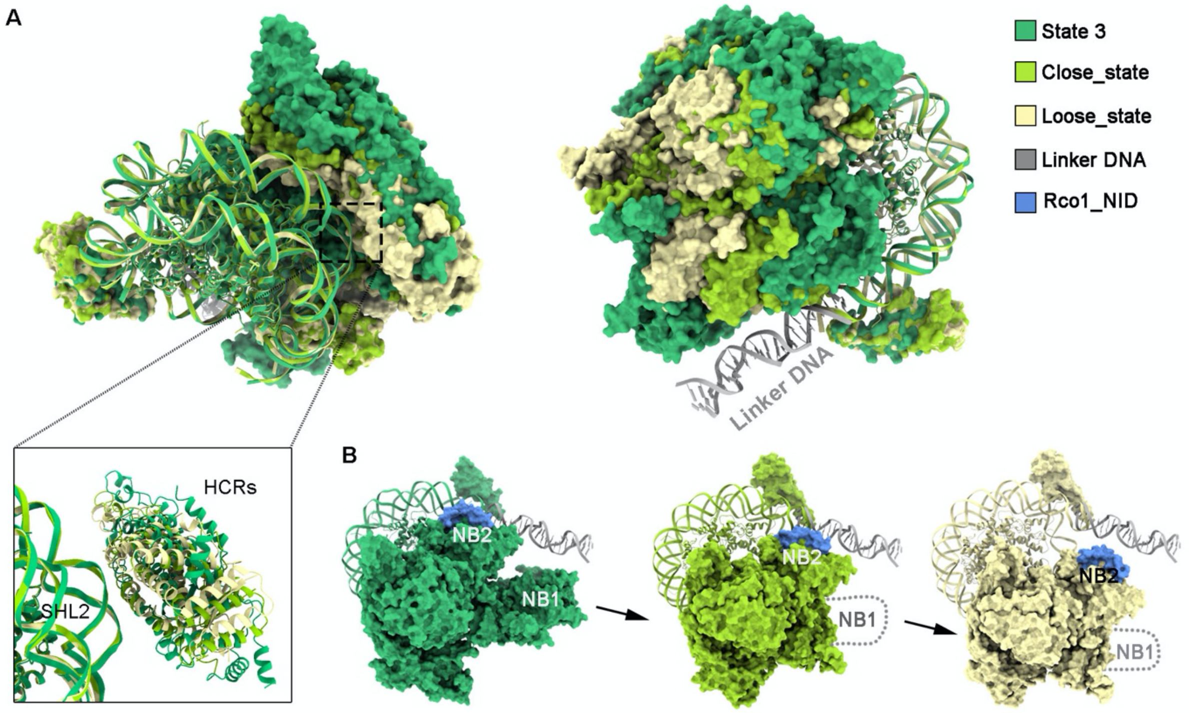

fig. S11 The linker DNA may mediate the Rpd3S working on neighbor nucleosomes. (**A**) Superposition of the nucleosome-bound Rpd3S complex in our State 3, Close_State (PDB code: 7YI4), and Loose_State (PDB code: 7YI5). The structure of linker DNA is predicted form the nucleosome structure with the PDB code 7VVU. Compared with recent report (*1*), our structure of State 3 exhibits similarities to our State 3 but slight alterations in orientation. Sequentially transitioning through State 3, Close_State, and Loose_State, the Rpd3S core exhibits a discernible motion of gradually sliding away from the nucleosome. The Sin3_HCRs anchor on the SHL2s. Nevertheless, the NB2 is sliding from SHL7 to the linker DNA; and the NB1 is moving away the nucleosome, likely leading to its flexibility and low resolution in Close/Loose_States. (**B**) The NB2 is sliding from SHL7 to the linker DNA; and the NB1 is moving away the nucleosome, likely leading to its flexibility and low resolution in Close/Loose_States. Previous reports revealed that the Rpd3S complex could bind di-nucleosome. It indicates that the linker DNA may mediate the Rpd3S working on neighbor nucleosomes.

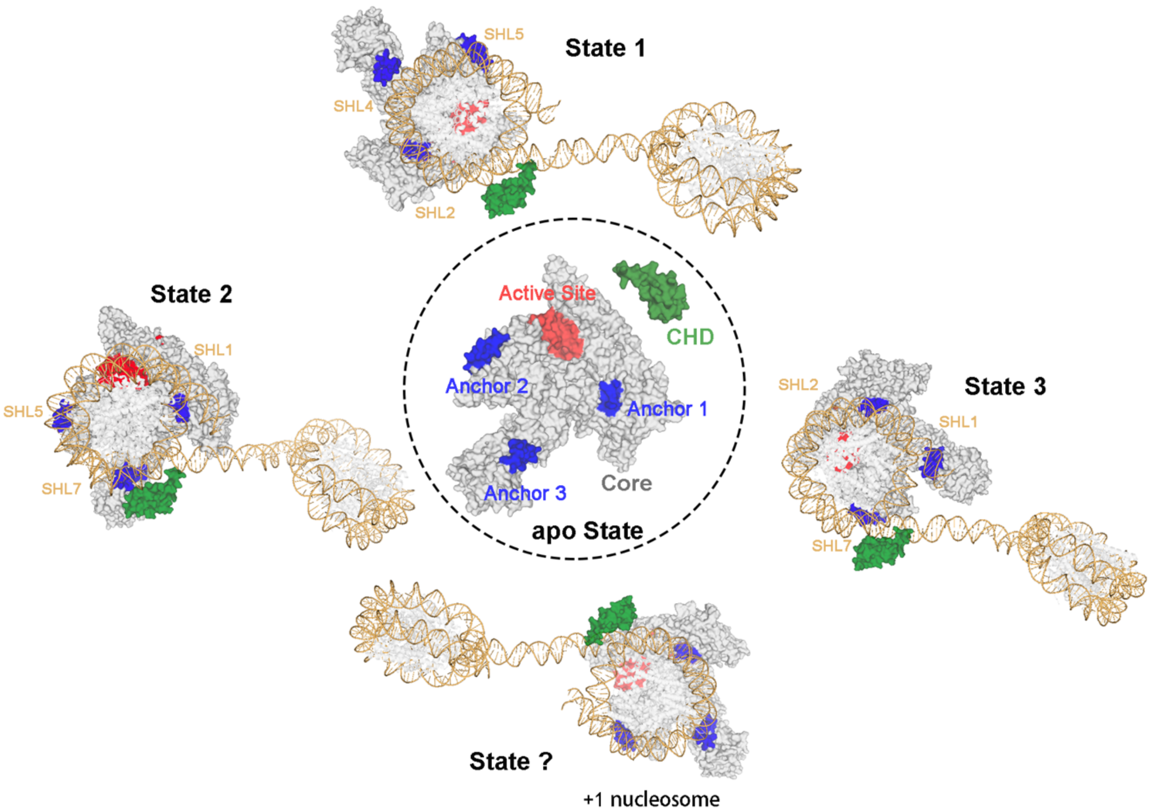

fig. S12 Working model for the Rpd3S sliding on the DNA track.
The structural analysis in this work points to a potential working model for the Rpd3S complex. In the apo state, the Eaf3_CHD is flexible relative to the core region of the Rpd3S complex. When recruited by the nucleosome with H3K36me marker, Eaf3_CHD would be stabilized at SHL7 during deacetylation. The Rpd3S core contains three positive-charged anchors, aiding to slide along the nucleosomal DNA to engage different deacetylation sites. Compared with recently reported structures, the extra linker DNA may mediate the Rpd3S to work on +1 nucleosome.

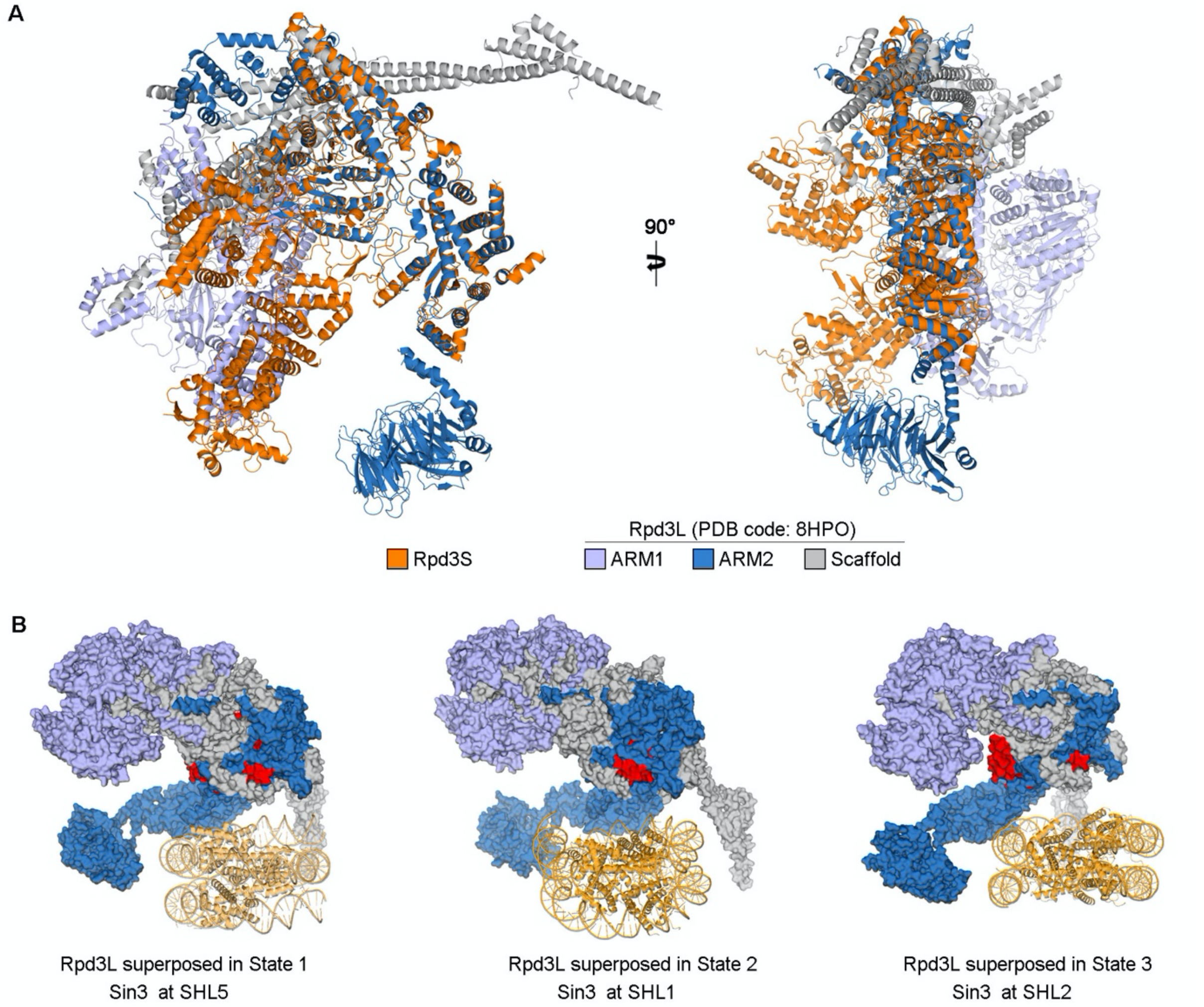

fig. S13 Comparison between *S. cerevisiae* Rpd3S and Rpd3L complexes.
(**A**) Superposition of the apo Rpd3S and Rpd3L (PDB code: 8HPO) complexes. The Rpd3L complex contains two Sin3 Lobes, named ARM1 and ARM2 respectively. The ARM1 is in the inhibited state with the active site of Rpd3 blocked, but the ARM2 is in an open conformation with the active site of Rpd3' exposed to the exterior space. The Sin3 Lobe of the Rpd3S is much similar with the ARM2 of the Rpd3L. (**B**) Superposition of the Rpd3L into the Rpd3S-nucleosome complex in three states. There is no clash between the Rpd3L and nucleosome in all states. The ARM2 of Rpd3L locates overhead the nucleosome, indicating its working mechanism; while the ARM1 is away from current nucleosome, suggesting its function on the extra linker DNA or -1/+1 nucleosome. The Rpd3 in ARM2 is highlighted in red.

table S1. Statistics of EM analysis and model validation.

	Rpd3S complex	Rpd3S-Nucleosome complex		
		State 1	State 2	State3
**Data collection**				
EM equipment	FEI Titan Krios	FEI Titan Krios		
Voltage (kV)	300	300		
Detector	K3	K3		
Pixel size (Å)	1.077	1.087		
Electron dose (e-/Å^2)	50	50		
Defocus range (μm)	1.8~2.3	1.8~2.3		
**Reconstruction**				
Software	cryoSPARC v4	RELION 3.0/cryoSPARC v4		
EMDB code	EMD-37364	EMD-37365/ EMD-37368/ EMD-37369	EMD-37366/ EMD-37370/ EMD-37371	EMD-37367/ EMD-37372/ EMD-37373
Number of particles	378,011	422,198/ 267,945/ 216,303	327,408/ 131,052/ 188,527	213,127/ 130,423/ 117,729
Symmetry	C1	C1		
Final masked resolution (Å)	3.3	3.9/2.9/2.9	3.6/3.6/2.9	4.4/3.9/3.2
Map sharpening B-factor (Å^2)	-170.4	-163.8/-114.3/ -101.8	-152.2/-123.7/ -100.3	-201.3/-129.5/ -100.6
**Model building**				
Software	Coot 0.8.9/Chimera	Coot 0.8.9/Chimera		
Refinement	phenix.real_space_refine	phenix.real_space_refine		
PDB code	PDB: 8W9C	PDB: 8W9D	PDB: 8W9E	PDB: 8W9F
Protein residues	1,868	3,005	2,991	2,990
B factors (Å^2)	55.01	73.96	88.90	75.62
**Validation**				
R.m.s deviations				
Bonds length (Å)	0.004	0.004	0.015	0.008
Bonds Angle (°)	0.660	0.693	0.784	0.701
Ramachandran plot statistics (%)				
Favored	93.64	94.57	93.54	94.90
Outlier	0.22	0.60	0.64	0.53
Clashscores	13.9	10.5	14.5	15.5
CaBLAM outliers (%)	3.6	3.2	2.8	2.5
MolProbity score	2.73	2.61	2.52	2.71

table S2. The distance measured from the active site to the N-terminal tailed-up residue of each histone in State 1/2/3, respectively.

	State 1			State 2			State 3		
	Tailed-up residue	Distance (Å)	Potential sites	Tailed-up residue	Distance (Å)	Potential sites	Tailed-up residue	Distance (Å)	Potential sites
**H3**	Pro38	89.5	-	Pro38	52.8	Lys14/18	Pro38	69.1	Lys14
**H3'**	Lys37	69.1	Lys14	Lys37	70.3	Lys14	Lys37	87.4	-
**H4**	Val21	52.0	-	Arg23	32.1	Lys5/8/12	Asp24	32.7	Lys5/8/12
**H4'**	Val21	82.1	-	Val21	88.8	-	Val21	93.3	-
**H2A**	Lys13	47.4	-	Ala12	80.0	-	Ala12	66.1	-
**H2A'**	Arg11	96.2	-	Arg11	102.1	-	Ala12	89.7	-
**H2B**	Lys30	65.9	-	Arg31	85.4	-	Arg31	80.4	-
**H2B'**	Lys30	74.8	-	Arg31	75.0	-	Arg31	58.6	-

*The HDAC activity on Lys14/18 of histone H3 and Lys5/8/12 of H4 for the Rpd3S complex has been verified in vitro.

Movie S1. The structure analysis reveals that the Rpd3S core likely rotates around an axle relative to the center of nucleosome.

Based on three nucleosome-bound structures, we used the "*Morph Conformations*" in *Chimera* to create a movie for mimicking trajectory, revealing that the Rpd3S core is rotating around an axle which relative to the center of the nucleosome. It may benefit the active center approach to different deacetylation sites in different histones.
